# Observations on the Management of Enteric Fever According to a Plan Based upon the So-called Specific Treatment

**Published:** 1883-03

**Authors:** James C. Wilson

**Affiliations:** Physician to the Jefferson Medical College Hospital, and to the Philadelphia Hospital


OBSEVATIONS ON THE MANAGEMENT OF ENTERIC FEVER ACCORDING TO A PLAN BASED UPON THE SO-CALLED SPECIFIC TREATMENT, By JAMES C. WILSON, M. D., Physician to the Jefferson Medical College Hospital, and to the Philadelphia Hospital. (Read January 3, 1883.)

I desire to lay before the College a plan of managing enteric fever, which I have employed during the past year and which, tested by such uncertain but not necessarily fallacious means as are available for a limited series of cases, has yielded satisfactory results.

The object of this communication will, I believe, be best attained by first sketching in outline the plan of treatment itself; next, by reviewing the considerations which led to its adoption, and finally by a brief study of the cases. This arrangement of the topics will enable us to economize time.

*The Plan of Treatment.*—The scope of this paper, and the necessity to be brief, debar me from the consideration of the general management of the patient, dietetics, the treatment of complications and sequels and of the prophylaxis; and restrict me, in the main, to the subject of the management by medicinal means. It is, in fact, this part of the treatment that, super-added to so-called rational and expectant method in general use in this community, differs from the common practice and constitutes the plan in question.

So soon as the patient is found to have enteric fever, or, in many instances, so soon as his symptoms warrant a reasonable suspicion that he is about to develop it, he is put to bed, ordered a diet consisting of milk, animal broths, jelly and simple custards, in small amounts, and at intervals of two or three hours. At night he is given a dose of calomel. This dose varies in amount from 7½ to 10 grains (0.5 to 0.66 gramme) and is repeated every second evening until three or rarely four doses have been administered in the course of the first six or eight days. It is given alone or in connection with sodium bicarbonate. There is commonly a slight increase of diarrhœa, if it be present, without aggravation of the other symptoms, and in some instances the tendency of the temperature at this time to steadily rise, appears to be controlled. If, as is frequently the case, spontaneous diarrhœa has not recurred in the first week, the calomel usually brings about two large evacuations on the day following its administration, not more. In either case the tendency to frequent passages in the later stages of the attack is favorably influenced by the repeated administration of this drug during the first week. If the case does not come under observation until after the tenth day, one only, or at most, two doses of calomel are given. No further doses of it are, however, given during the course of the attack unless constipation occurs. In this event, if the evidences of extensive or deep implication of the intestinal wall, such as abdominal pain, tenderness or marked tympany are absent, calomel in 7½-grain (0.5 gramme) doses is given at intervals of three or four days. If there is reason to suspect serious intestinal lesions, the lower bowel may be more safely emptied of its contents every third or fourth day, by enemata of moderate size (8 to 10 fluid-ounces). It is necessary to bear in mind that the gravest lesion of the gut leading even to hæmorrhage and perforation, have occasionally been observed in cases characterized, not only by constipation, but also by an entire absence of pain or tenderness, and very moderate tympany. The danger of salivation from calomel in these doses in enteric fever appears to be slight. In only one case in sixteen were the mercurial fetor and slight swelling of the gums observed.

Excessive diarrhœa has been controlled by the use of opium, either in suppositories, containing 1 grain (0.06 gramme), or by the mouth in quarter-grain (0.016 gramme) doses, often associated with bismuth, and given *pro re nata*. It is an invariable rule that the patient be kept in the horizontal position and to the use of the bed-pan and urinal, from the time of the recognition of the disease until defervescence is completed. He is, however, turned upon his side from time to time, and made to maintain that position for twenty or thirty minutes, if necessary, being supported by the nurse.

From the beginning of the attack the following mixture is regularly administered in doses of one, two, or even three drops in a sherry-glassful of ice-water after food, every two or three hours during the day and night:

| | | |
|---|---|---|
| ℞. | Tinct. iodinii, fʒij....................8 | 00 c. c. |
| | Acid. carbolici liq., fʒj.............4 | 00 c. c. |
| | M. | |

Unless some unusual circumstance occur to render a change necessary, this medicine is not suspended until the attack draws to a close. It is well borne by the stomach and excites no repugnance on the part of patients. In one case only has it been necessary to omit the carbolic acid on account of the disgust assumed by its odor.

Partly for the sake of its favorable influence upon the skin and for the sake of cleanliness, partly because of its favorable though slight influence upon the temperature, the patient is to be sponged twice a day with equal parts of aromatic vinegar or alcohol, and cold water. If it is more grateful to him, this sponging may be done in tepid water, the evaporation of an extensive film of water not below the temperature of his body probably being not without a refrigerating tendency.

When the evening axillary temperature reaches 104° F. (40° C.) quinine in massive doses, 24 to 30 grains (1.66 to 2.00 grammes) is given upon a falling temperature. I usually direct 8 to 10 grains to be given in solution at 5, at 5.30, and at 6 A. M. the following morning. Administered thus at the decline of the temperature in its diurnal revolution, these large doses of quinine depress it from 2.5° to 3.5° F. (1.4° to 1.8° C.). After the lapse of forty-eight to seventy-two hours, if necessary the dose may be repeated. If these doses be rejected by the stomach—an unusual circumstance—half the quantity of quinine may be administered hypodermically. For this purpose a citric acid solution is to be preferred. Since the adoption of the plan of treatment under consideration, I have not encountered cases attended with such hyperpyrexia as has rendered attempts to control it by cold baths necessary or even advisable.

The minor nervous symptoms are best held in check by skillful nursing. For the relief of the headache of the first ten days, absolute quietude, a dim light, etc., are often sufficient; occasionally the bromides alone, or in combination with chloral are required. Later in the course of the disease chloral is unsafe. From the end of the first week the patient cannot be left unattended even for a few minutes, without risk. Persons in whom delirium was only occasional and transient, have in many instances destroyed themselves during the momentary absence of the nurse.

Alcohol is not often indicated prior to the beginning of the third week. It may, however, by reason of the habits of certain patients, be necessary throughout the attack. Although forming no essential part of the treatment, it is commonly administered in varying though usually small amounts towards the close of the sickness. Some patients do well without taking it at all. It is of course administered in accordance with well-understood indications upon the supervention of delirium, ataxic symptoms and the evidences of failures of the forces of the circulation. The patients are carefully watched well into convalescence, and cautioned against too soon regarding themselves restored to health.

The dangers of the establishment of a focus of contagion are guarded against by the systematic, thorough disinfection of the stools immediately after they are voided.

The considerations which induced me to adopt this plan of treatment indicated in the foregoing sketch, are:

1. A feeling of dissatisfaction regarding the expectant method of treating enteric fever. This feeling, vague at first, grew more definite and stronger with increasing clinical opportunities, and a fuller knowledge of the natural history of the disease, until it became a motive, impelling me to cast about for some different and more satisfactory plan. This feeling has been during the past decade, a very general one in the profession in all parts of the world, as is attested by an almost endless succession of journal articles setting forth new plans of treatment, and the use of new drugs in the management of this, the most common and most important of the acute infectious diseases of the present epoch in medical history. Most of the plans thus suggested have led to disappointment when tested by the fuller observations of the profession; many of them have failed to attract general attention, and some few are still *sub judice*. Their number and diversity bear witness to a wide-spread distrust of the once well-established expectant treatment. This distrust is, however, based upon something more tangible than a mere feeling of dissatisfaction. The statistics of all observers whose cases have been sufficiently numerous to be trustworthy, show enteric fever to be, when treated by the expectant plan, a disease of high death-rate.

The percentage of fatal cases rarely falls below 15 per cent., and often exceeds 25 per cent., according to the hospital records of this country, Great Britain, and Continental Europe. Jaccoud, with a collection of 60,000 cases, observed a mortality of 20 per cent.; Murchison, in 27,051 cases, 17.45 per cent.; Leibermeister, in 1,718 cases, at Basle, under an expectant plan, records 27.3 per cent. of deaths. But turning from broad generalizations to personal experience, who is there here that, many times elated by the happy issue of mild or average cases treated by the expectant plan, has not realized the sense of utter powerlessness attending it when he has stood face to face with cases in which *to do* rather than *to wait*, has been necessary to save life?

2. Enteric fever is the very type of the general diseases, of affections *totius substantiæ*. The tissues are universally implicated in the morbid proccsses; no function of the body wholly escapes perturbation. For this reason, plans of treatment suggested by the prominence of certain groups of symptoms, or by the known lesions of particular organs, even though of undoubted benefit as far as they go, are in theory unsatisfactory, because they are directed in effect against conspicuous manifestations of the cause of the sickness rather than against the cause itself.

Whilst in actual practice, the treatment by turpentine, by alcohol, by opium with lead, or the silver nitrate, or by agents capable of controlling the febrile movement, as quinine, digatalis, salicin, and the salicylates, even the cold-water treatment itself, although at times, and in the hands of certain clinicians showing favorable results—all these have failed of general acceptance on the part of the profession.

3. The general character of the disease, the specific nature of its cause, the unsatisfactory results alike of an expectant and of a symptomatic plan of treatment, or rather of the two combined, have united to render the idea of a specific treatment, a true cure for enteric fever, a most attractive one, to stimulate thoughtful observers to renew again and again the disappointing search for it. To this idea may be traced the treatment by the mineral acids, by chlorine-water, by carbolic acid, by quinine alone, by quinine and digitalis, by iodine, by the potassium iodide, by calomel.

4. No only is the conception of a specific treatment for specific diseases a most attractive one, and the attainment of such a treatment for enteric fever brought within the bounds of a reasonable hope by the analogy of syphilis and the malarial diseases, but the search after it with due caution and judgment, has also the warrant of the very highest medical authority.

Passing by some earlier names, I refer to Da Costa, who has said: "It would be as illogical as absurd to suppose that we shall never possess the coveted means really to cure the continued fevers. Doubtless, to the physicians of the time of Charles V., the radical and specific treatment of the malarial fevers appeared as hopeless and remote as the radical and specific treatment of the continued fevers appears to the scientific inquirer of our day."

I refer also to Liebermeister, who, treating about 800 cases, part with calomel, part with iodine, had, with the former drug, a mortality of only 11.7 per cent., with the latter of 14.6 per cent., against 18.3 per cent. for cases treated without those remedies, but in other respects upon a similar plan.

Bartholow has also spoken in favorable terms of the treatment by iodine in combination with carbolic acid.

The treatment adopted is thus seen to consist of the use of the two remedies that are proved to exert a favorable influence upon the disease, iodine and calomel, with the addition of carbolic acid in minute amounts. I am aware that no positive conclusions as to the efficacy of particular plans of treatment can be deduced from a limited series of cases. I am also aware that few acute diseases show greater variations in intensity and in the percentage of mortality at different periods, and under different circumstances, than enteric fever. Nevertheless, I have ventured to occupy your attention with this subject to-night, because the results of the treatment encourage me to hope that its discussion in this way will lead to its trial on a more extended scale. That it amounts to a specific treatment in the narrow sense is not affirmed. It is tentative, provisional, but it is, nevertheless, to be regarded as a contribution to the subject of the specific treatment of enteric fever.

The total number of cases treated by this plan is sixteen; all recovered, one being now in the second week of convalescence.

Of these, eight were severe, the temperature reaching or exceeding 104° F. (40° C.).

Of these eight severe cases, one was characterized by uncontrollable vomiting in the third week. The patient retained no food taken by the mouth for five consecutive days.

One case was very irregular in its course, and was complicated by an obscure abdominal abscess which discharged by the bowel. The temperature in this case on two occasions attained 105° F. (40.5° C.). This case presented the characteristic eruption of enteric fever.

A third case was prolonged by a severe relapse.

Of the eight cases in which the observed temperature did not at any time attain 104° F. (40° C.), and which was therefore looked upon as medium or mild cases, one was complicated by crural phlebitis, and another by the occurrence of intestinal hæmorrhage.

The average duration of the eight severe cases was about 31 days; that of the eight mild and medium cases was about 25 days.

Of the whole number ten were treated in hospital, six in private practice. All from the time of their coming under observation were under my personal care.

In two cases the special plan of treatment was abandoned about the beginning of the third week on account of the supervention of unusual symptoms of great gravity. These related respectively to gastric irritability and an obscure abdominal abscess.

These sixteen cases are unfortunately not a consecutive series. During the year in which I have had the opportunity of observing them, two other cases of enteric fever have occurred in my hospital practice in which this plan of treatment was not employed. One was that of a man suffering from rheumatism, who, after a stay of several weeks in the wards, and in a bed near that occupied by a patient very ill of enteric fever, was observed to be febrile, and to have the typhoid eruption. This person, previously greatly reduced, was not regarded as a suitable subject for a special treatment, the efficacy of which was not yet established in my mind. The other was a man, who, with an obscure history of a sickness of many weeks, and a very irregular temperature, developed the typhoid eruption, and within forty-eight hours had general peritonitis. These two fatal cases have however no bearing upon the result of the treatment.

In private practice several cases of mild continued fever of long duration were treated upon this plan during the past winter. I believe them to have been anomalous cases of enteric fever, but as the rose spots of that disease were absent, and their departure from the typical disease was wide, I have not included them in the above collection of cases treated. They all recovered.

The result of this plan of treatment has not only been satisfactory in respect of the recovery of all the cases treated, an accidental circumstance not liable to mislead persons familiar with the disease, but it has also been satisfactory in respect of the general course of the attack, and the appearance of the patient. These were in the main, despite the severe type of the disease, in several of the cases and despite the occurrence of grave complications, favorable. I make this statement with due regard to the personal equation, and with no willingness to permit the observed fact to differ from the actual fact, for I desire any who may make trial of this plan to be more favorably impressed with the results of it, than they have been impressed with my account of it.

[After the reading of the preceding paper :—]

Dr. George Hamilton spoke of the great importance of preventing hypostatic congestion by changing the position of the patient from time to time. This was one feature of the plan of treatment recommended some time ago by Dr. Wm. Pepper, in typhoid fever, by which he obtained the unequalled result of 98 per cent. of recoveries. He was not, however, at this time able to recall in detail the method of treatment.

Dr. J. T. Eskridge stated that the treatment to which Dr. Hamilton referred consisted in the administration of nitrate of silver, and was that which had been introduced by the late Dr. J. K. Mitchell some years ago.

Dr. Roberts Bartholow said that the plan of treatment of typhoid, advocated in the very interesting and able paper just read, is, as all present probably know, in part, the so-called "specific" method. The administration of calomel in full purgative doses during the first week serves a double purpose: it has an effect on the range of temperature, and it acts on the typhoid germs present and multiplying in the intestinal canal. The use of iodine—usually Lugol's solution—throughout the disease, is also one mode of the specific treatment. By the use of this medicine, it is attempted to prevent the multiplication of germs in the intestine, to check fermentation, and to maintain an antiseptic action in the blood.

Although the existence of typhoid germs has not been proved, it must be regarded as possible. Klein, a few years ago, announced the discovery of the specific organism of typhoid in the affected intestinal glands, but Creighton of Cambridge, showed that the supposed germs were produced by the mode of preparation. This *fiasco* threw great discredit on the whole question of germs. Nevertheless, the course of treatment directed against supposed germs—the antiseptic method—has had a most favorable influence on the progress and mortality from typhoid. Whilst the specific plan has been advocated in Germany, the Montpelier school have brought forward carbolic acid as the remedy, and the success which has attended the use of this remedy has been really remarkable. Quite a different complexion has been put on the statistics of mortality since they began the use of carbolic acid.

It is probable that the combination of carbolic acid and iodine gives better results than the use of either singly. According to my observation, this method of treatment diminishes the diarrhœa, lowers the fever, and renders the disease much less violent, consequently lessening the mortality. Dr. Wilson has, therefore, rendered us a real service by drawing attention anew to this plan of medication, and especially by supporting his position with valuable cases and statistics. Besides this use of medicines, Dr. Wilson's treatment contains many valuable suggestions and practical methods, which, no doubt, contribute materially to his success.

Dr. J. M. Da Costa spoke of the purgative treatment in enteric fever, as that which has been tried in the French Hospitals and for a time sanctioned by Louis. As regards calomel it was partly by its purgative action that it was supposed to be beneficial. In his hands the calomel treatment had not yielded favorable results. He had found carbolic acid useful in controlling diarrhœa and in lowering the temperature. He had also employed thymol in one-half to one grain doses. He suggested the use of this remedy in the place of carbolic acid, as more acceptable to the stomach.

Dr. Wilson, in reply to the question of Dr. Hamilton, said that he considered it necessary to frequently change the position of the patient to prevent pulmonary hypostasis. He had intended to emphasize this point in his paper. He called attention to the fact that carbolic acid and like drugs probably exert a favorable influence upon the course of enteric fever by their power to stay the rapid decomposition of the intestinal contents, which, for lack of the antiseptic influence of the intestinal juices, the bile, etc., all of which are changed, is a secondary cause of irritation, diarrhœa and tympany. Calomel also, he thought, probably exerted an indirect beneficial influence in the same direction.

---

Dr. H. M. Mayer has been commissioned by Governor Cullom to make an official report on his return from Europe, upon foreign lunacy management.

